# Supplementary material for: Evaluation of Two Different CMV-Immunoglobulin Regimens for Combined CMV Prophylaxis in High-Risk Patients following Lung Transplant
Source: Microorganisms. 2022 Dec 22;11(1):32. doi: 10.3390/microorganisms11010032 (PMC9864217; doi:10.3390/microorganisms11010032)
Supplement: Supplementary file 1 [file microorganisms-11-00032-s001.zip › microorganisms-2098132-supplementary.pdf]

Evaluation of two different CMV-immunoglobulin regimens for combined CMV prophylaxis in high-risk patients following lung transplant

Supplementary Materials

TABLE OF CONTENTS		PAGE
**Table S1**	Risk factors for first CMV infection after the end of the antiviral prophylaxis (valganciclovir) using Cox regression	2
**Figure S1**	Kaplan-Meyer analysis of the time to first CMV infection A) stratified by whether the patient discontinued the antiviral prophylaxis prematurely and B) by CMV-Ig schedule among those who completed 12 months of antiviral prophylaxis	3
**Figure S2**	Kaplan-Meier curve of the cumulative risk of first CMV infection.	4

Table S1. Risk factors for first CMV infection after the end of the antiviral prophylaxis (valganciclovir) using Cox regression.

Variables		Crude HR (95% CI)	P-value	Adjusted HR (95% CI)	P-value
**Age at transplant**		0.993 (0.979–1.008)	0.364	**-**	-
**Months of VGCV prophylaxis**		0.979 (0.934–1.028)	0.395		
**Immunosuppression induction**					
	Yes	1.448 (0.828–2.352)	0.194		
	No	Ref.			
**Indication for lung transplant**					
	COPD	Ref.			
	DILD	1.021 (0.577–1.808)	0.943	0.841 (0.397-1.781)	0.650
	Bronchiectasis	0.997 (0.547–1.818)	0.993	0.869 (0.397-1.898)	0.724
	PAH	**2.814 (1.356–5.838)**	**0.005**	**4.155 (1.528-11.300)**	**0.005**
	Other	0.753 (0.326–1.741)	0.507	0.960 ((0.395-2.336))	0.929
**Acute rejection**		1.003 (0.769–1.309)	0.981		
**m-TOR inhibitor**					
	Yes	1.011 (0.656-1.558)	0.959		
	No	Ref.			
**Premature VGCV prophylaxis discontinuation**					
	Yes	1.319 (0.842–2.064)	0.227		
	No	Ref.			
**CMV-Ig schedule**					
	SR-Ig	Ref.			
	ER-Ig	0.948 (0.622–1.446)	0.805		

CMV, cytomegalovirus; COPD, chronic obstructive pulmonary disease; ER-Ig, extended CMV-Ig; DILD, diffuse interstitial lung disease; GVC, ganciclovir; SR-Ig, label use or short CMV-Ig regimen; OR, odds ration; PAH, pulmonary arterial hypertension; VGCV, valganciclovir.

Figure S1. Kaplan-Meyer analysis of the time to first CMV infection A) stratified by whether the patient discontinued prematurely the antiviral prophylaxis and B) by CMV-Ig schedule among those who completed 12 months of antiviral prophylaxis.

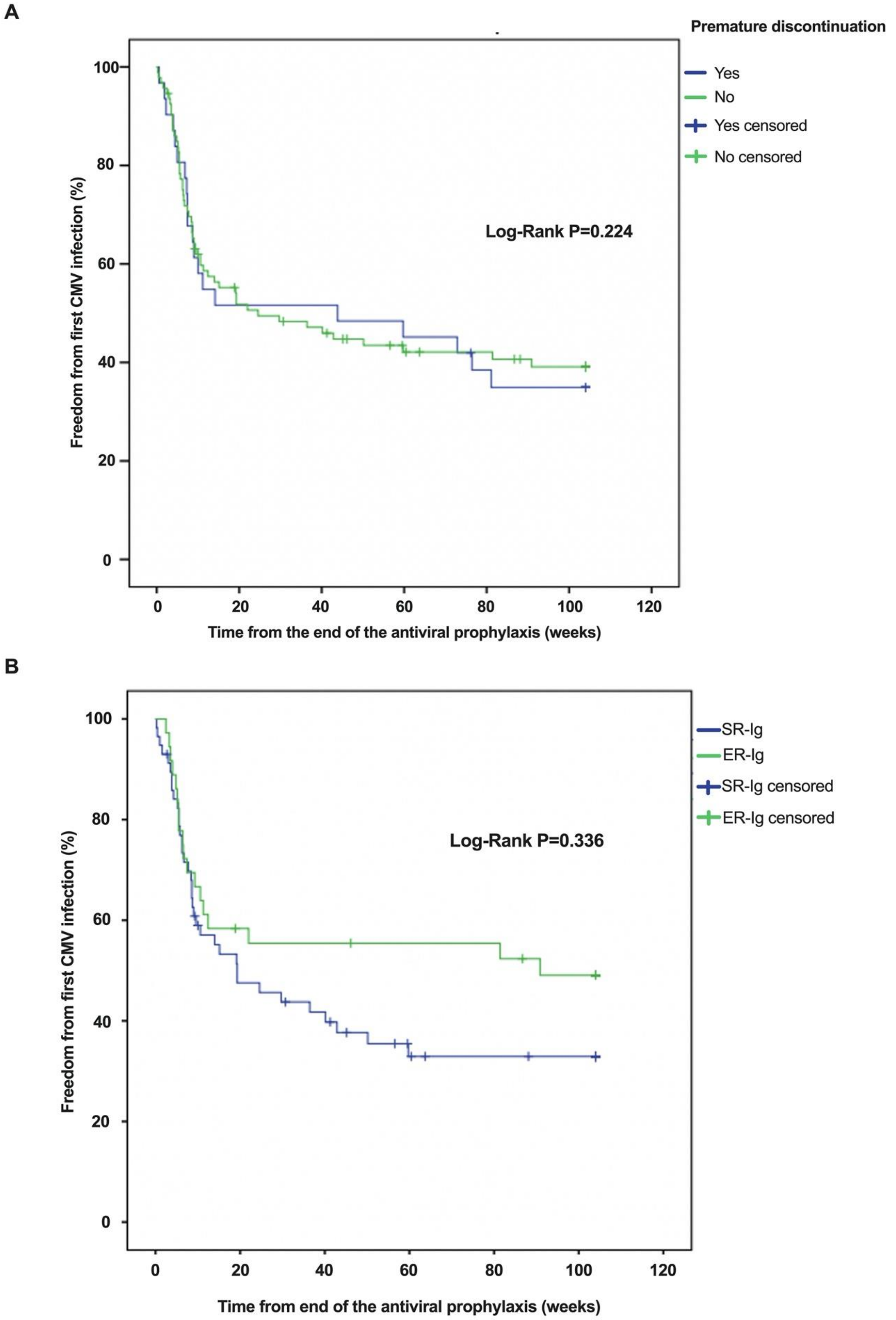

CMV, cytomegalovirus; ER-Ig, extended CMV-Ig; SR-Ig, label use or short CMV-Ig.

Figure S2. Kaplan-Meier curve of the cumulative risk of first CMV infection.

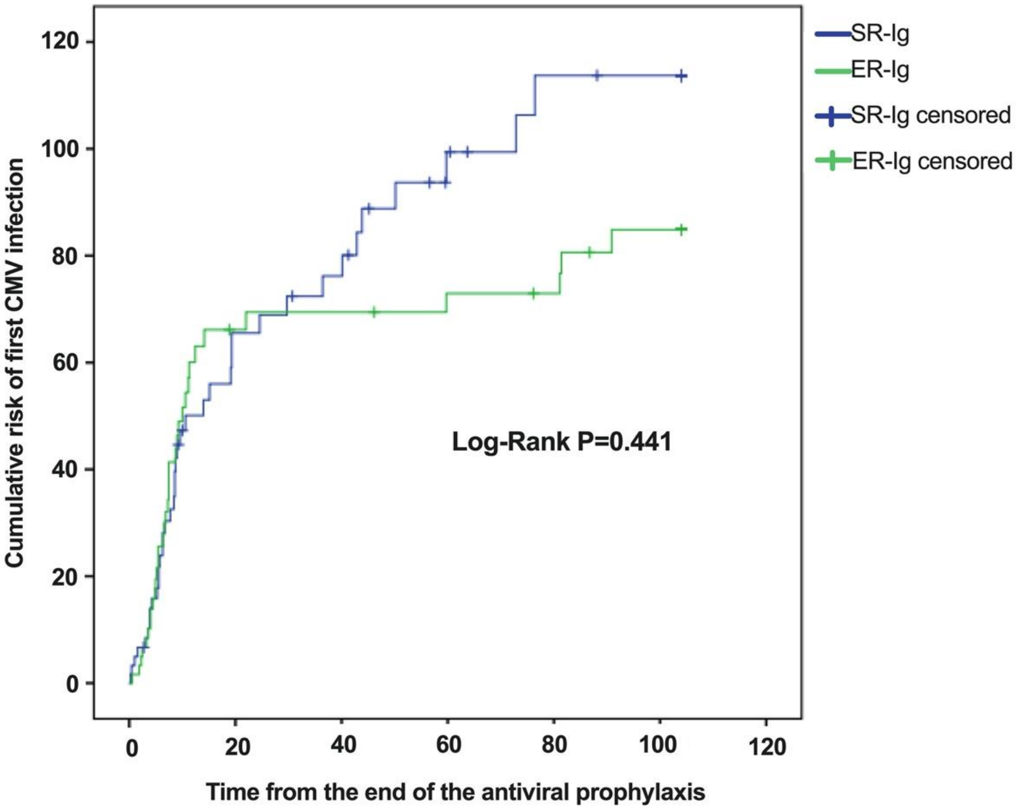

CMV, cytomegalovirus; ER-Ig, extended CMV-Ig; SR-Ig, label use or short CMV-Ig.
